# High-efficiency solution-processed OLED based on trivalent europium complex by modifying the composition of the multiple-host system

**DOI:** 10.3389/fchem.2022.965927

**Published:** 2022-09-14

**Authors:** Xiaofang Li, Jiaxuan Yin, Jingyu Wang, Ruixia Wu, Shuaibing Li, Weidong Sun, Liang Zhou

**Affiliations:** ^1^ State Key Laboratory of Rare Earth Resource Utilization, Changchun Institute of Applied Chemistry, Chinese Academy of Sciences, Changchun, China; ^2^ School of Applied Chemistry and Engineering, University of Science and Technology of China, Hefei, China

**Keywords:** organic light-emitting diodes, solution-processed method, multiple-host system, host materials, recombination zone

## Abstract

In this work, di-[4-(N,N-ditolylamino)-phenyl]cyclohexane (TAPC); 4,4′,4″-tri (9-carbazoyl)triphenylamine (TcTa); 9-(4-tert-butylphenyl)-3,6-bis(triphenylsilyl)-9H-carbazole (CzSi); and 1,3,5-tri (m-pyrid-3-yl-phenyl)benzene (TmPyPB) were used to constitute the multiple-host system and fabricate solution-processed organic light-emitting diodes (s-OLEDs) with europium complex Eu(DBM)_3_Phen (DBM, 1,3-diphenylpropane-1,3-dione; Phen,1,10-phenanthroline) as emitter. In order to determine the optimal composition of the multiple-host system, a series of devices with different light-emitting layers (EMLs) were fabricated and compared. Experimental results revealed that removing TmPyPB out of the multiple-host system greatly reduces the turn-on voltage, whereas the addition of TcTa to the multiple-host system helps facilitate the transfer of holes from TAPC to Eu(DBM)_3_Phen molecules, thus increasing the recombination probability of carriers on emitter molecules. Finally, high performance solution-processed red OLED (turn-on voltage of 3.8 V) based on the europium complex doped multiple-host system obtained the maximum current efficiency of 2.07 cd A^−1^, power efficiency of 1.54 lm W^−1^, external quantum efficiency of 1.2%, and brightness of 945 cd m^−2^.

## Introduction

Lighting and display productions have become indispensable parts of human life in recent years. Among them, organic light-emitting diodes (OLEDs) have emerged as competitive candidates because of their excellent performances. The emitters included in the light-emitting layer (EML) of OLEDs determine emission color, radiative energy transition, electrical modulation, and other photophysical processes of devices (Gong et al., 2012; Wu and Zhu, 2013; Xu et al., 2014). By profiting from the shielding effect of 5s^2^5p^6^ to 4f orbital, lanthanide complexes possess unique narrow emissions from the *f-f* transition (Bunzli and Piguet, 2005). Among the lanthanide complexes, tris-*β*-diketonate europium (III) (Eu^3+^) complexes perform high color purity with a sharp emission peak at 612 nm, originating from the ^5^D_0_→^7^F_2_ transition of Eu^3+^ (Kido et al., 1994; Liang et al., 2000). In addition, both singlet and triplet energies of ligands can transfer to the center ions of lanthanide complexes. Therefore, they can achieve 100% quantum efficiency theoretically (Wang et al., 2019). Accordingly, Eu^3+^ complexes have enormous potential as emitters of OLEDs.

However, the characteristic transition of ^5^D_0_→^7^F_2_ is Laporte forbidden for free Eu^3+^ ion. This leads to a long lifetime of about several hundred microseconds in the excited state of Eu^3+^ systems (Halverson et al., 1964; D’Aléo et al., 2012). As a result, a high concentration of excited states caused by their long lifetime is the main reason for triplet–triplet annihilation (TTA), which leads to the low efficiency of OLEDs based on europium complexes (Pereira et al., 2019).

Compared with traditional vacuum-evaporated OLEDs (v-OLEDs), solution-processed organic light-emitting diodes (s-OLEDs) have the advantages of low-cost and simple operation (Yook and Lee, 2014). However, it is more convenient to realize multi-layer v-OLEDs because each layer is stacked sequentially through programmed temperature-controlled thermal evaporation. Therefore, v-OLEDs have the characteristics of no damage to underlying film and uniform film coverage (He et al., 2004). Moreover, the typically encountered inter-diffusion and intermixing problems make it difficult to realize multi-layer s-OLEDs, so using cross-linkable materials or orthogonal solvent are feasible methods to fabricate multi-layer s-OLEDs (Aizawa et al., 2014).

In this work, s-OLEDs with the typical structure consisting of solution-processed hole injection layer (HIL) and EML, as well as the vacuum-deposited electron transporting layer (ETL) and cathode, were fabricated. In addition, the dependence of electroluminescent (EL) properties on the composition of the multiple-host system with Eu(DBM)_3_Phen as emitter was investigated, whereas the composition of the multiple-host system was optimized in detail. Finally, di-[4-(N,N-ditolylamino)-phenyl]cyclohexane (TAPC):4,4′,4″-tri (9-carbazoyl)triphenylamine (TcTa):9-(4-tert-Butylphenyl)-3,6-bis(triphenylsilyl)-9H-carbazole (CzSi) with the ratio of 2:5:1 was determined to be the optimal multiple-host system. The corresponding device obtained the maximum current efficiency (CE) of 2.07 cd A^−1^, power efficiency (PE) of 1.54 lm W^−1^, external quantum efficiency (EQE) of 1.2%, and brightness of 945 cdm^−2^. More importantly, the turn-on voltage of this device is as low as 3.8 V.

### Experiments

All the organic materials used in this study were obtained commercially and used as received (purity >99%). The patterned indium-tin-oxide (ITO) substrates were degreased with a detergent, rinsed in ultra-purified water, and then dried at 120°C for 1 h in an oven. The cleaned patterned ITO substrates (10 Ω/sq) were subsequently treated with oxygen plasma for 20 min to improve the work function. After that, the poly(3,4-ethylenedioxythiophene):poly(styrene sulfonate) (PEDOT:PSS, Clevios P VP Al 4083) layer with a thickness of 32 nm was deposited onto the pre-cleaned ITO substrate through spin-coating and annealed at 120°C for 20 min in air. EML was spin-coated onto the PEDOT:PSS layer with chlorobenzene solution at 3,000 r/min for 30 s and annealed at 70°C for 30 min within a glove box with a nitrogen atmosphere. Among them, aqueous dispersions of PEDOT:PSS were filtered through a 0.45 μm syringe filter, and the solutions of materials used in EML were filtered through a 0.22 μm syringe filter. After that, ETL was grown at the rate of 0.05 nm s^−1^ under high vacuum (≤3.0 × 10^−5^ Pa) in a vacuum chamber. In another vacuum chamber (≤8.0 × 10^−5^ Pa), LiF and Al layers were deposited at the rate of 0.01 and 1 nms^−1^, respectively. Without being exposed to the atmosphere. The thicknesses of these deposited layers and the evaporation rate of individual materials were monitored in vacuum conditions with quartz crystal monitors. The overlap zone of ITO and Al electrodes with the area of 3 × 3 mm were the active emitting dots.

A Keithley 2000 multi-meter and a Keithley 2400 source meter with a silicon photodiode were used to measure the current density-brightness-voltage (*J-B-V*) characteristics, and the EL spectra were collected with an Ocean Optics spectrophotometer. The absorption spectra were measured with a Thermo NanoDrop 2000c spectrophotometer, whereas the photoluminescent (PL) spectra were recorded on a Hitachi F-7000 fluorescence spectrophotometer. The EQE of the EL device was calculated by brightness, EL spectrum, and current density passing through the device. For measurement of the transient EL spectra, a Transient EL Measurement System McScience M6200 was used.

## Results and discussion

**FIGURE 1:**
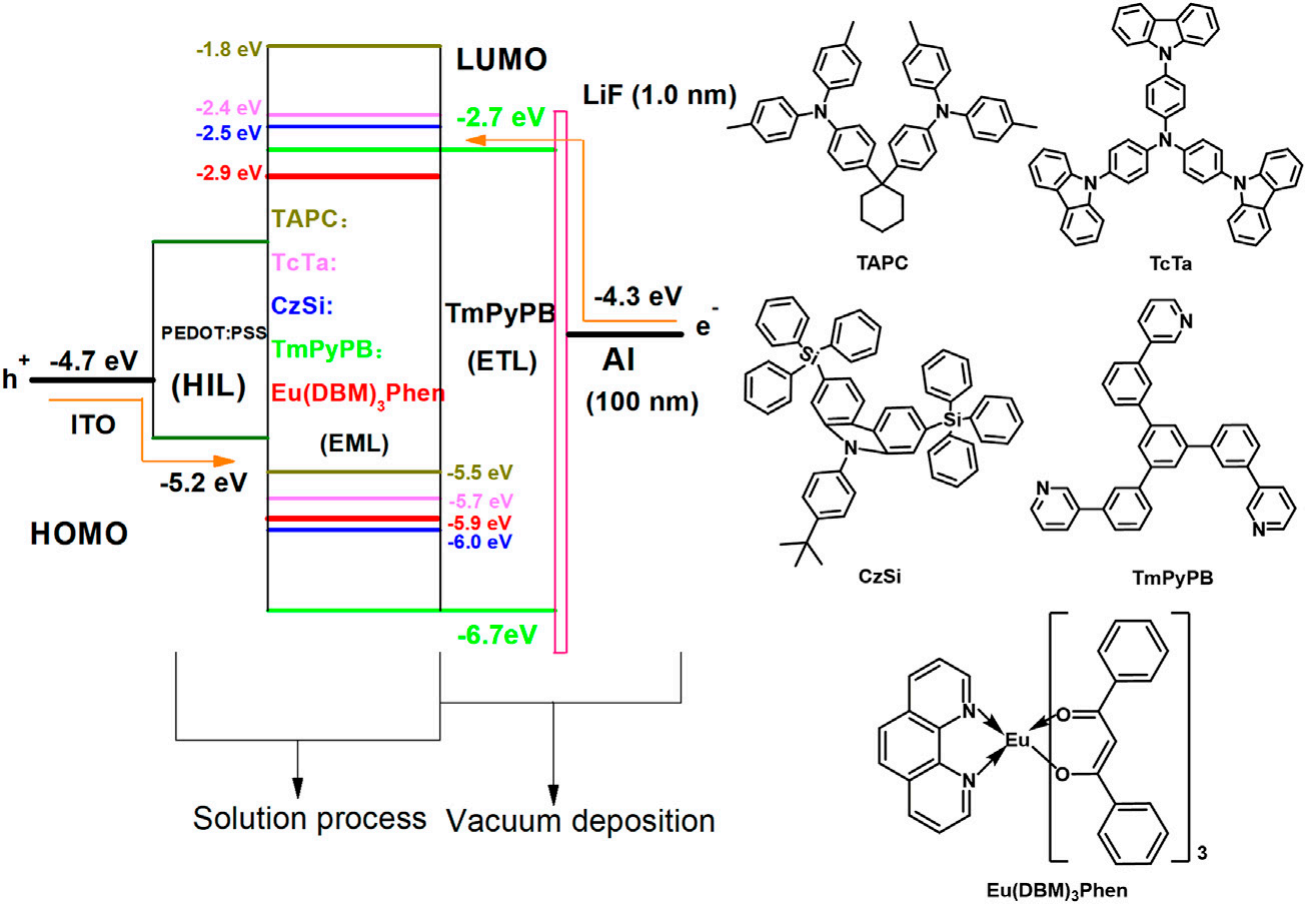
Proposed energy levels’ diagram of the designed OLEDs and the molecular structures of TAPC, TcTa, CzSi, TmPyPB, and Eu (DBM)_3_Phen.

The structure and energy level diagram of the initially designed device is shown in Figure 1, whereas the basic structure of the designed device is ITO/PEDOT:PSS (32 nm)/EML/TmPyPB (60 nm)/LiF (1 nm)/Al (100 nm). In this case, the classical water-soluble PEDOT:PSS was routinely used as the HIL material. However, insufficient hole injection and unsatisfactory electron/exciton blocking effect at the PEDOT:PSS/EML interface will significantly influence the performances of the designed s-OLEDs, which have two solution-processed films (HIL and EML) and three vacuum deposition films (ETL and cathode) (Kim et al., 2005). Therefore, to improve the performances of s-OLEDs, it is necessary to choose an appropriate host and ETL materials. Then, TAPC, TcTa, CzSi, and 1,3,5-tri (m-pyrid-3-yl-phenyl)benzene (TmPyPB) were used as host materials due to their individual characteristics. TAPC could well confine the triplet excitons within EML due to its high triplet energy. Furthermore, its high hole mobility (1 × 10^−2^ cm^2^ V^−1^ s^−1^) and high-lying lowest unoccupied molecular orbital (LUMO) level help facilitate hole transport and block electrons (Lee et al., 2008). TcTa plays the role of hole transport material as well because its moderate highest occupied molecular orbital (HOMO) level helps accelerate the transfer of holes from HIL into EML although its hole mobility (3.1 × 10^−4^ cm^2^ V^−1^ s^−1^) is lower than that of TAPC (Kang et al., 2007). Besides, bipolar material CzSi and electron transport material TmPyPB were used as host materials to broaden the recombination zone. What is more, TmPyPB also acts as the hole block and electron transport layer material because of its high electron mobility (1 × 10^−3^ cm^2^ V^−1^ s^−1^) and low-lying HOMO level (−6.7 eV) (Su et al., 2008).

**FIGURE 2:**
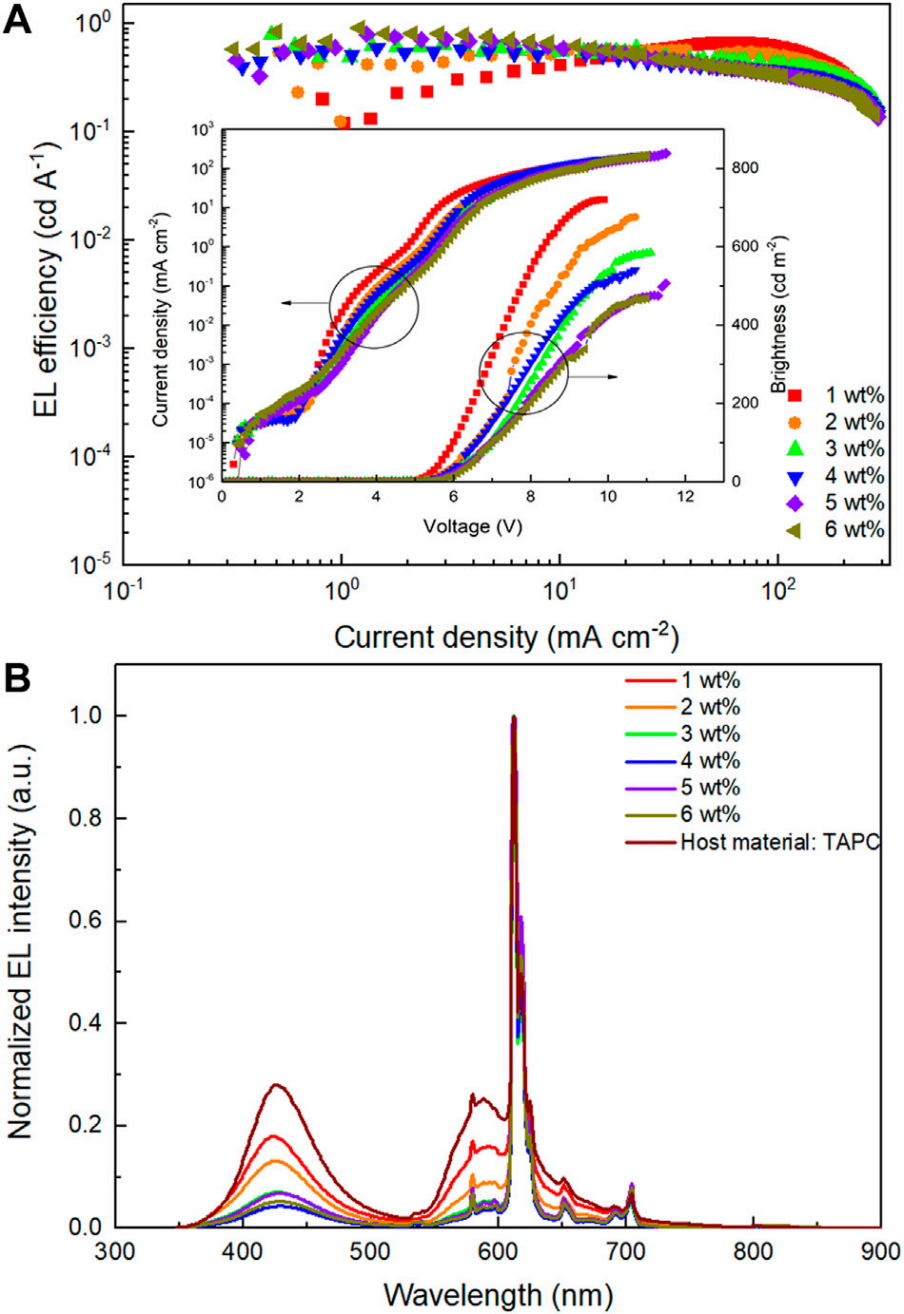
**(A)** EL efficiency-current density (*η-J*) characteristics of devices with Eu(DBM)_3_Phen at different concentrations. Inset: current density-brightness-voltage (*J-B-V*) characteristics of devices with Eu(DBM)_3_Phen at different concentrations. **(B)** Normalized EL spectra of devices with Eu(DBM)_3_Phen at different concentrations and devices possessing host material only TAPC operating at 10 mA cm^−2^.

To optimize the doping concentration of Eu(DBM)_3_Phen within EML, six devices with Eu(DBM)_3_Phen (x wt%):TAPC:TcTa:CzSi:TmPyPB (2:2:1:1) film as EML were fabricated by controlling x to be 1, 2, 3, 4, 5, and 6, respectively. As shown in Figure 2A, with increasing doping concentration, EL efficiency increased gradually at low current density while decreasing at high current density basically. As a result, the devices with doping concentrations between 2 and 4 wt% got a smooth change of current efficiency. As shown in Figure 2B, the EL spectra of multiple-host devices operating at 10 mA cm^−2^ were compared with that of the device with only TAPC as the host material. Besides the characteristic emission peak at 612 nm, another emission peak at 427 nm was also observed. Here, the 427 nm emission originated from excitons generated on TAPC molecules. In addition, TAPC emitted more intensely in relatively lower doping concentrations such as 1 and 2 wt%, thus causing the color of these devices to be pink at 10 mA cm^−2^ although these devices displayed higher brightness. In this case, the doping concentration of Eu(DBM)_3_Phen was identified as 4 wt% because the device with a doping concentration of 4 wt% (device M) obtained the best comprehensive performance combining efficiency and color purity.

**FIGURE 3:**
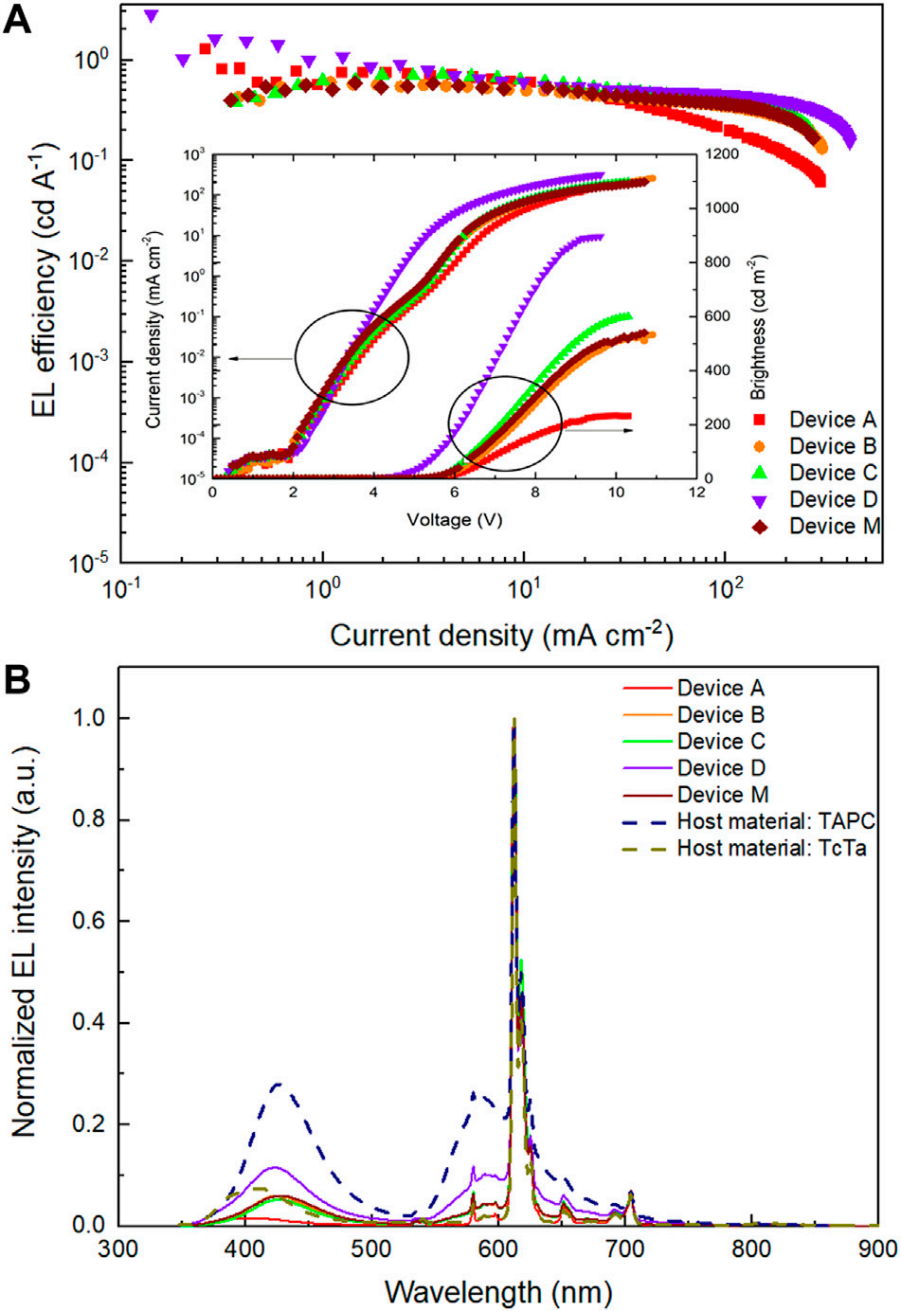
**(A)** EL efficiency-current density (*η-J*) characteristics of devices A, B, C, D, and M. Inset: current density-brightness-voltage (*J-B-V*) characteristics of devices A, B, C, D, and M. **(B)** Normalized EL spectra of devices A, B, C, D, and M and devices possessing host material only TAPC or TcTa operating at 10 mA cm^−2^.

Due to the multiple-host materials in EML, it was necessary to clarify the influence of each host material on device performances and thus confirm the optimal multiple-host composition. Therefore, four devices based on three hosts system with EML consist of Eu(DBM)_3_Phen (4 wt%):TcTa:CzSi:TmPyPB (2:1:1) (device A); Eu(DBM)_3_Phen (4 wt%):TAPC:CzSi:TmPyPB (2:1:1) (device B); Eu(DBM)_3_Phen (4 wt%):TAPC:TcTa:TmPyPB (2:2:1) (device C); and Eu(DBM)_3_Phen (4 wt%):TAPC:TcTa:CzSi (2:2:1) (device D), respectively, which were fabricated and investigated. Compared with other devices, as depicted in Figure 3A and Table 1, the device without TmPyPB (device D) displayed fantastic performances with dramatically reduced turn-on voltage and increased current intensity at the equivalent applied voltage. These could be ascribed to the low-lying energy levels of TmPyPB, which led to abundant electrons on TmPyPB molecules because the low concentration of Eu(DBM)_3_Phen causes the saturation of electrons on Eu(DBM)_3_Phen molecules although Eu(DBM)_3_Phen has even lower LUMO level compared with TmPyPB. As a result, unbalanced carriers’ distribution caused the higher turn-on voltage and lower current efficiency of the devices with TmPyPB as one of the host materials. Besides, the addition of TAPC also has a significant impact on device brightness. In this case, it is easy for TAPC molecules to trap holes due to the high-lying HOMO level of TAPC, so the emergence of TAPC emission at low current density means the presence of electrons on TAPC molecules, which is also caused by the saturation of electrons on Eu(DBM)_3_Phen molecules as well as the accumulation of electrons within EML.

**TABLE 1:** The key properties of devices A, B, C, D, and M.

Device	V_turn-on_ (V)	B^a^ (cd m^−2^)	ƞ_c_ ^b^ (EQE^c^) (cd A^−1^)	ƞ_p_ ^d^ (lm W^−1^)	CIE_x, y_ ^e^
A	5.1	234	0.76 (0.4%)	0.43	(0.640, 0.316)
B	5.0	533	0.60 (0.4%)	0.35	(0.487, 0.247)
C	5.1	601	0.72 (0.4%)	0.39	(0.548, 0.279)
D	4.0	895	1.62 (0.9%)	1.21	(0.455, 0.244)
M	4.9	541	0.59 (0.4%)	0.34	(0.521, 0.268)

a The data for maximum brightness (B).

b Maximum current efficiency (ƞ_c_).

c Maximum external quantum efficiency (EQE).

d Maximum power efficiency (ƞ_p_).

e Commission Internationale de l'Eclairage coordinates (CIE_x, y_) at 10 mA cm^−2^.

**FIGURE 4:**
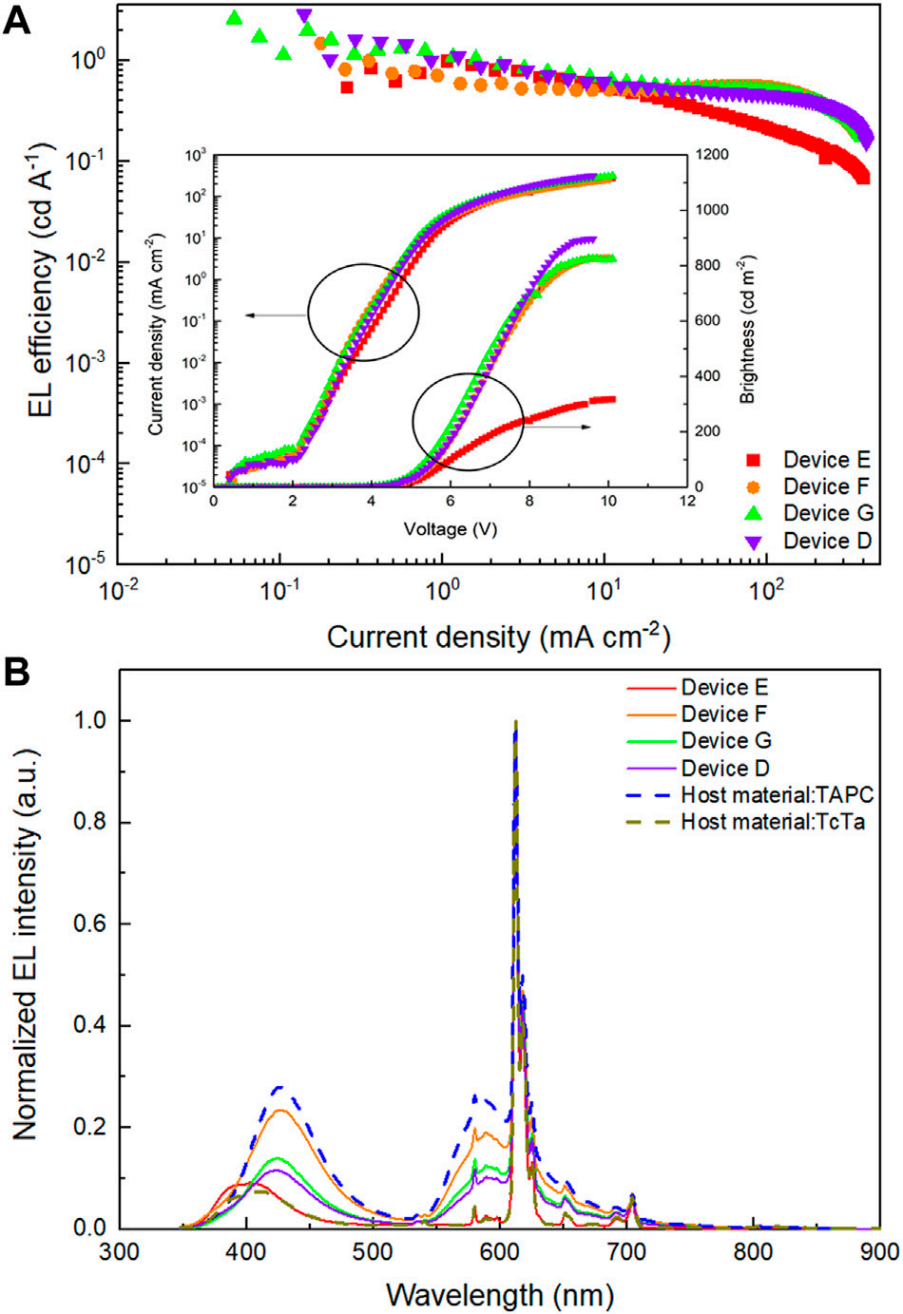
**(A)** EL efficiency-current density (*η-J*) characteristics of devices E, F, G, and D. Inset: current density-brightness-voltage (*J-B-V*) characteristics of devices E, F, G, and D. **(B)** Normalized EL spectra of devices E, F, G, and D and devices possessing host material only TAPC or TcTa operating at 10 mA cm^−2^.

**FIGURE 5:**
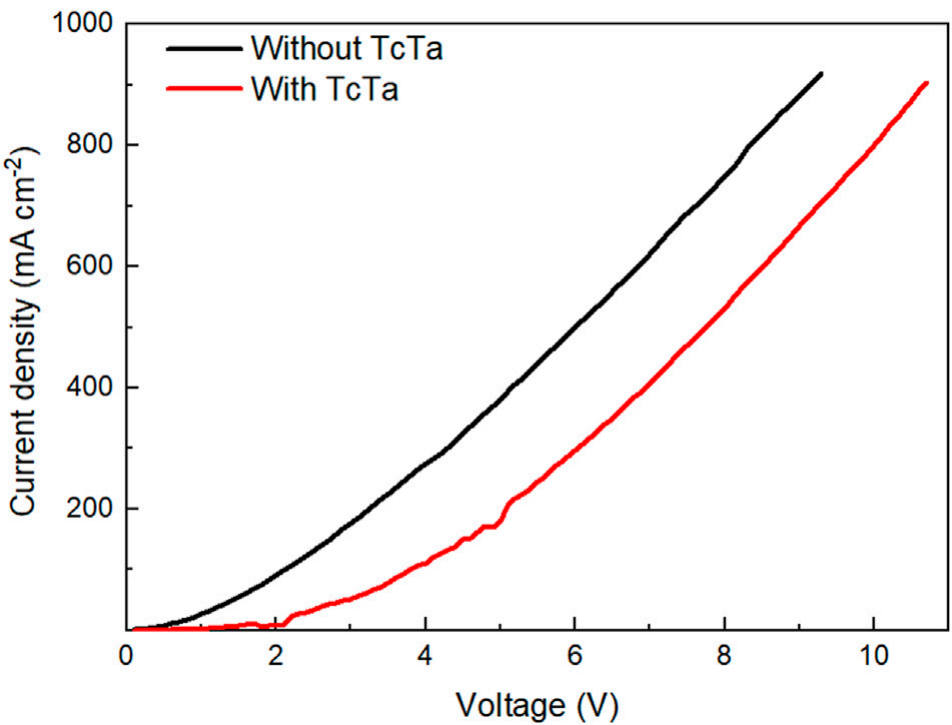
Current density-voltage (*J-V*) characteristics of the hole-only devices with TcTa or without TcTa doped in EML.

Based on device D, to determine the optimal composition of the multiple-host system, three devices with EML of Eu(DBM)_3_Phen (4 wt%):TcTa:CzSi (2:1) (device E); Eu(DBM)_3_Phen (4 wt%):TAPC:CzSi (2:1) (device F); and Eu(DBM)_3_Phen (4 wt%):TAPC:TcTa (1:1) (device G), respectively, were further designed and fabricated. As shown in Figure 4B and Table 2, although the performances of device G were slightly better than those of device D, device D possessed better color purity and Commission Internationale de l'Eclairage coordinates. What is more, compared with device D, a noticeable decrease in current efficiency was found in device F for the absence of TcTa. To gain insight into the impact of TcTa, as shown in Figure 4A, two hole-only devices composed of ITO/PEDOT:PSS (32 nm)/Eu(DBM)_3_Phen (4 wt%):TAPC:TcTa:CzSi (2:x:1)/HAT-CN (10 nm)/Al (100 nm) (x = 0, 2) were fabricated and measured. The J-V curves of these two hole-only devices are depicted in Figure 5. With the addition of TcTa, the current density-voltage curve shifted toward high voltage, which means the delay of hole transport. A wider recombination zone would be realized in the device with the addition of TcTa because the delayed hole transport caused the shift of the recombination center toward the anode. In this case, the delay of hole transport had something to do with the relatively lower hole mobility of TcTa compared with TAPC. Theoretically speaking, as shown in Figure 1, the presence of TcTa molecules helps facilitate the transfer of holes from TAPC to Eu(DBM)_3_Phen molecules because of the well-matched HOMO level of TcTa, which is between those of TAPC and Eu(DBM)_3_Phen.

**TABLE 2:** The key properties of devices E, F, G, and D.

Device	V_turn-on_ (V	B^a^ (cd m^−2^)	ƞ_c_ ^b^ (EQE^c^) (cd A^−1^)	ƞ_p_ ^d^ (lm W^−1^)	CIE_x, y_ ^e^
E	4.4	317	0.97 (0.6%)	0.63	(0.548, 0.306)
F	3.9	828	0.99 (0.6%)	0.76	(0.358, 0.191)
G	3.6	826	1.97 (1.1%)	1.58	(0.425, 0.228)
D	4.0	895	1.62 (0.9%)	1.21	(0.455, 0.244)

a The data for maximum brightness (B).

b Maximum current efficiency (ƞ_c_).

c Maximum external quantum efficiency (EQE).

d Maximum power efficiency (ƞ_p_).

e Commission Internationale de l'Eclairage coordinates (CIE_x, y_) at 10 mA cm^−2^.

**FIGURE 6:**
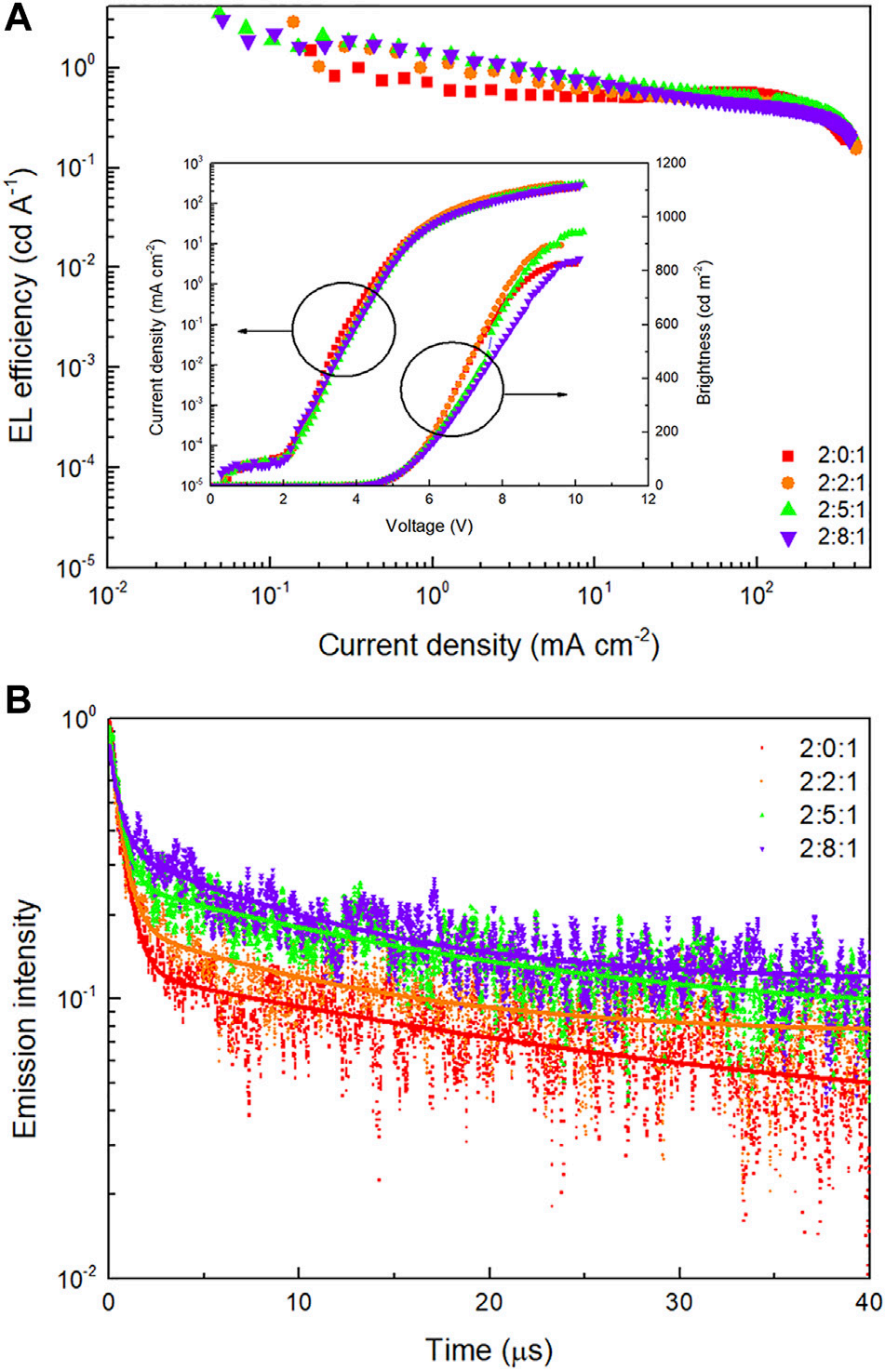
**(A)** EL efficiency-current density (*η-J*) characteristics of devices with TAPC:TcTa:CzSi at different ratios. Inset: current density-brightness-voltage (*J-B-V*) characteristics of devices with TAPC:TcTa:CzSi at different ratios. **(B)** Transient EL decay curves of devices with TAPC:TcTa:CzSi at different ratios and corresponding biexponential fitting curves.

In addition, to evaluate the influence of TcTa on device properties, a series of devices with EML of Eu(DBM)_3_Phen (4 wt%):TAPC:TcTa:CzSi (2:x:1, x = 0, 2, 5, 8) were fabricated and compared. The best properties were obtained when the ratio of host materials was 2:5:1 (device H). As shown in Figure 6A and Table 3, higher EL efficiency demonstrated the improved carriers’ balance on Eu(DBM)_3_Phen molecules. Generally, the long excited state lifetime of europium complexes causes severe TTA, thus leading to rapid roll-off of EL efficiency (Adachi et al., 2000). As depicted in Figure 6B, with increasing addition of TcTa, increasing excited state lifetime indicates the broadening recombination zone, which helps suppress exciton annihilation. According to previous studies, TTA and charge charrier imbalance seemed to be the principle mechanisms resulting in efficiency roll-off in phosphorescent OLEDs (Murawski et al., 2013). According to the exponential function to quantify the decay in Figure 6B, biexponential decay had a better fit to four sets of transient decay data than monoexponential decay. Therefore, TTA might be a decaying way for triplet excitons except for their phosphorescent emission. Besides, the emission of ^5^D_1_ state from Eu^3+^ at 540 nm (^5^D_1_ → ^7^F_1_) and 589 nm (^5^D_1_ → ^7^F_3_) approximately in all EL spectra (Figure 3B) illustrated the existence of TTA (Canzler and Kido, 2006). The process of TTA could well explain the state as follows:

$$T_{1}+T_{1}\overset{k_{TT}}{\to }T_{n}\left(S_{n}\right)+S_{0}\to T_{1}\left(S_{1}\right)+S_{0}.$$

**TABLE 3:** The key properties of devices with TAPC:TcTa:CzSi at different ratios.

Device	V_turn-on_ (V)	B^a^ (cd m^−2^)	ƞ_c_ ^b^ (EQE^c^) (cd A^−1^)	ƞ_p_ ^d^ (lm W^−1^)	CIE_x, y_ ^e^
2:0:1	3.9	828	0.99 (0.6%)	0.76	(0.358, 0.191)
2:2:1	4.0	895	1.62 (0.9%)	1.21	(0.455, 0.244)
2:5:1	3.8	945	2.07 (1.2%)	1.54	(0.474, 0.256)
2:8:1	3.8	845	1.86 (1.1%)	1.36	(0.511, 0.272)

a The data for maximum brightness (B).

b Maximum current efficiency (ƞ_c_).

c Maximum external quantum efficiency (EQE).

d Maximum power efficiency (ƞ_p_).

e Commission Internationale de l'Eclairage coordinates (CIE_x, y_) at 10 mA cm^−2^.

Here, T_1_, S_1_, and S_0_ represent the triplet, singlet, and ground state, respectively. T_n_ and S_n_ mean the high-lying state of triplet and singlet, $k_{TT}$ is the rate constant for the kinetics of the TTA process (Murawski et al., 2013). ^5^D_1_ state is a higher excited state compared to the ^5^D_0_ state. As a result, TTA was a critical element leading to the roll-off in this study, and we could find that the transient decay became increasingly curved with decreasing proportion of TcTa, obviously revealing a proportion-dependent quenching of Eu^3+^ triplet.

**FIGURE 7:**
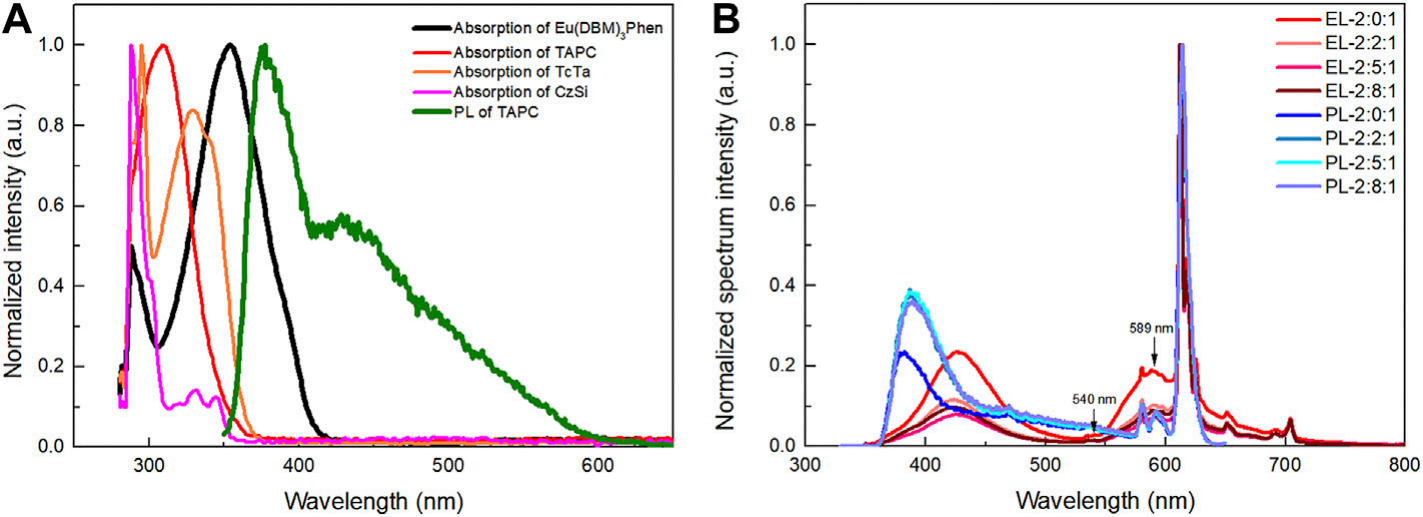
**(A)** Absorption spectra of Eu(DBM)_3_Phen, TAPC, TcTa, CzSi in chlorobenzene solution, and PL spectrum of TAPC film. **(B)** Normalized EL spectra of devices with TAPC:TcTa:CzSi at different ratios operating at 10 mA cm^−2^ and normalized PL spectra of Eu(DBM)_3_Phen (4 wt%):TAPC:TcTa:CzSi (TAPC:TcTa:CzSi = 2:x:1, x = 0, 2, 5, and 8, respectively) films.

Carriers’ trapping and Förster energy transfer were previously identified as the two main EL mechanisms of doped devices (Uchida et al., 1999; Blumstengel et al., 2001). Which mechanism existed or dominated relies on the dopant and host materials in the system. As described in Figure 7A, the marked overlap between the TAPC fluorescence spectrum and Eu(DBM)_3_Phen absorption spectrum indicates that Förster energy transfer from TAPC to Eu(DBM)_3_Phen is possible. What is more, conspicuously, the TAPC fluorescence spectrum only overlapped with Eu(DBM)_3_Phen absorption spectrum, which means that Förster energy transfer only existed from TAPC to Eu(DBM)_3_Phen. After that, as depicted in Figure 7B, the PL spectra of the films with composition of Eu(DBM)_3_Phen (4 wt%):TAPC:TcTa:CzSi (TAPC:TcTa:CzSi = 2:x:1, x = 0, 2, 5, 8) were measured under the excitation of 308 nm and compared with EL spectra of aforementioned devices operating at 10 mA cm^−2^. The addition of TcTa caused enhanced TAPC emission in the PL spectra, whereas TAPC emission in EL spectra decreased gradually with increasing the TcTa ratio. This result further proved the broadening recombination zone with the addition of TcTa, which caused more Eu(DBM)_3_Phen molecules to participate in the EL processes and thus the increased recombination of carriers on Eu(DBM)_3_Phen molecules instead of TAPC molecules. In other words, carriers’ trapping is the dominant EL mechanism of these devices. Moreover, the weaker emission of ^5^D_1_ state in EL illustrated the decrease in TTA within the appearance of TcTa, which demonstrated that TcTa could broaden the recombination zone effectively.

## Conclusion

In conclusion, we have designed and solution-processed the red-emitting devices based on trivalent europium complex Eu(DBM)_3_Phen by regulating the composition of host materials within EML. On account of the slower hole mobility of TcTa compared with TAPC, the presence of TcTa molecules delayed the transport of holes within EML to some degree and facilitated the transfer of holes from TAPC to Eu(DBM)_3_Phen molecules, thus resulting in broadening recombination zone and improved carriers balance on Eu(DBM)_3_Phen molecules. Therefore, enhanced efficiency was realized due to the raised recombination probability of carriers on Eu(DBM)_3_Phen molecules. Finally, the optimal device with a turn-on voltage of 3.8 V obtained the maximum current efficiency of 2.07 cd A^−1^, power efficiency of 1.54 lm W^−1^, external quantum efficiency of 1.2%, and brightness of 945 cd m^−2^.

## Data Availability

The original contributions presented in the study are included in the article. Further inquiries can be directed to the corresponding author.
